# Biodegradation of variable-chain-length *n*-alkanes in *Rhodococcus opacus* R7 and the involvement of an alkane hydroxylase system in the metabolism

**DOI:** 10.1186/s13568-014-0073-4

**Published:** 2014-09-30

**Authors:** Jessica Zampolli, Elena Collina, Marina Lasagni, Patrizia Di Gennaro

**Affiliations:** 1 Department of Biotechnology and Biosciences, University of Milano-Bicocca, Piazza della Scienza 2, Milano, 20126, Italy; 2 Department of Earth and Environmental Sciences, University of Milano-Bicocca, Milano, Italy

**Keywords:** Rhodococcus, n-alkanes degradation, Alkane hydroxylase, AlkB, Enzymatic expression

## Abstract

*Rhodococcus opacus* R7 is a Gram-positive bacterium isolated from a polycyclic aromatic hydrocarbon contaminated soil for its versatile metabolism; indeed the strain is able to grow on naphthalene, *o*-xylene, and several long- and medium-chain *n*-alkanes. In this work we determined the degradation of *n*-alkanes in *Rhodococcus opacus* R7 in presence of *n-*dodecane (C12), *n-*hexadecane (C16), *n-*eicosane (C20), *n-*tetracosane (C24) and the metabolic pathway in presence of C12. The consumption rate of C12 was 88%, of C16 was 69%, of C20 was 51% and of C24 it was 78%. The decrement of the degradation rate seems to be correlated to the length of the aliphatic chain of these hydrocarbons. On the basis of the metabolic intermediates determined by the R7 growth on C12, our data indicated that *R. opacus* R7 metabolizes medium-chain *n*-alkanes by the primary alcohol formation. This represents a difference in comparison with other *Rhodococcus* strains, in which a mixture of the two alcohols was observed. By GC-MSD analysis we also identified the monocarboxylic acid, confirming the terminal oxidation.

Moreover, the *alkB* gene cluster from *R. opacus* R7 was isolated and its involvement in the *n*-alkane degradation system was investigated by the cloning of this genomic region into a shuttle-vector *E. coli*-*Rhodococcus* to evaluate the alkane hydroxylase activity. Our results showed an increased biodegradation of C12 in the recombinant strain *R. erythropolis* AP (pTipQT1-*alk*R7) in comparison with the wild type strain *R. erythropolis* AP. These data supported the involvement of the *alkB* gene cluster in the *n*-alkane degradation in the R7 strain.

## 1 Introduction

The problems associated with contaminated sites are assuming rising prominence in many countries (Vidali, [2001]), due to the increase of anthropogenic pollution in the environment. Anthropic activities produce a wide amount of pollutants, such as aliphatic and aromatic polycyclic hydrocarbons; soil and water microorganisms can selectively degrade these xenobiotic compounds as the only carbon and energy source. The knowledge of the metabolic pathways of these microorganisms allows the reclamation of polluted sites. There are a lot of studies on the metabolism of these compounds in Gram-negative bacteria while Gram-positive bacteria have not been investigated to the same extent. Members of the *Rhodococcus* genus, found in many environmental niches, have a marked ability to metabolize a wide variety of xenobiotic compounds, and it is well recognized that species of this genus are key participants in the recycling of complex organic compounds (Finnerty, [1992]; Larkin et al., [2005]; Martínková et al., [2009]). The genetic rearrangement and plasticity of the *Rhodococcus* genome have appeared to play a significant role in its adaptation to a wide variety of environmental contaminants (Leahy and Colwell, [1990]; Van der Meer et al., [1992]; Kulakov et al., [1998]; Larkin et al., [2006]; McLeod et al., [2006]).

Alkane degraders are bacteria that have a very versatile metabolism, so that they can use as carbon source many other compounds in addition to alkanes (Smits et al., [2002]; Margesin et al., [2003]; Harayama et al., [2004]). The ability of these bacteria of degrading *n*-alkanes is well established, but relatively little is known about the molecular characteristics of their alkane-degradative systems.

Alkanes are saturated, linear molecules whose chain length can vary from 1 (in methane) to more than 50 carbon atoms. They are the major components of petroleum fuels which can be commonly found in contaminated environments (So and Young, [1999]), indeed they constitute about 20-50% of crude oil, depending on the source of the oil. In addition, alkanes (predominantly long-chain compounds) are produced throughout the biosphere by living organisms (plants, algae and bacteria) as a waste product, a structural element, a defense mechanism, or as a chemoattractant (van Beilen et al., [2003]). The aerobic degradation of these molecules starts by oxidation of one of the terminal methyl groups to generate the corresponding primary alcohol by alkane hydroxylases (AHs). The oxidation can occur on various positions: terminal or subterminal, to final conversion to a fatty acid (van Beilen et al., [2003]; Ji et al., [2013]). So far some alkane-degradative systems of a small number of Gram-negative bacteria have been well characterized, such as those of *Alkanivorax, Pseudomonas* and *Acinetobacter* (Alonso and Roujeinikova, [2012]; Ratajczak et al., [1998]; van Beilen and Funhoff, [2007]; Wang and Shao, [2013]). The *alk* system found in *Pseudomonas putida* GPo1, which degrades *n*-alkanes from *n*-pentane to *n*-dodecane, remains the most extensively characterized alkane hydroxylase system. It is a three-component alkane hydroxylase complex consisting of a particulate nonheme integral membrane alkane monooxygenase (AlkB) and two soluble proteins, rubredoxin (AlkG) and rubredoxin reductase (AlkT) (Alonso and Roujeinikova, [2012]).

Much less is known about the alkane-degradative systems of Gram-positive bacteria. Homologs of *alkB* gene were amplified from psychrotrophic *Rhodococcus* sp. Q15 (Whyte et al., [2002]), *Gordonia* sp. strain SoCg (Lo Piccolo et al., [2011]) and *Rhodococcus opacus* B4 (Sameshima et al., [2008]).

The *Gordonia* sp. strain SoCg unique *alkB* gene was analyzed by functional heterologous expression in *E. coli* and in *S. coelicolor,* which were shown to oxidize *n*-hexadecane to the correspondent primary alcohol, 1-hexadecanol, but no expression occurred in presence of long-chain *n*-triacontane. Similarly, the *Rhodococcus opacus* B-4 *alkB1* and *alkB2* genes were expressed heterologously in two *E. coli* recombinants which were able to convert *n*-alkanes (*n*-pentane to *n*-hexadecane) to their corresponding alcohols in anhydrous organic solvents. The start codons of two genes were changed from GTG to ATG to express them in *E. coli. Rhodococcus* sp. strain Q15 was shown to be able to mineralize *n*-dodecane and *n*-hexadecane and bioconvert them into corresponding primary and secondary alcohols at 5°C. The utilization of potential metabolic intermediates indicated that Q15 oxidizes alkanes by both the terminal oxidation pathway and the subterminal oxidation pathway (Whyte et al., [1998]).

*Rhodococcus opacus* R7 is a Gram-positive bacterium isolated from a polycyclic aromatic hydrocarbon contaminated soil for its ability to grow on naphthalene. It is characterized by a versatile metabolism, indeed the strain is able to grow on naphthalene, *o*-xylene, and several long- and medium-chain *n*-alkanes. In previous studies, the genes involved in the degradation of naphthalene and *o*-xylene were identified, and the related metabolic pathways were characterized (Di Gennaro et al., [2010]). In this work we determined the degradation of *n*-alkanes in *Rhodococcus opacus* R7, performing biodegradation kinetics in presence of *n-*dodecane (C12), *n-*hexadecane (C16), *n-*eicosane (C20), *n-*tetracosane (C24), and the metabolic pathway in presence of C12. Moreover, the *alkB* gene cluster from *R. opacus* R7 was isolated and its involvement in *n*-alkane degradation system was investigated by cloning and expression of this genomic region.

## 2 Materials and methods

### 2.1 Bacterial strains, growth conditions and general procedures

Bacterial strains and plasmids used in this study are listed in Table 1. *Rhodococcus opacus* R7 (CIP 107348), isolated for its ability to grow on naphtalene and *o*-xlyene (Di Gennaro et al., [2001]), was grown on M9 mineral medium (Maniatis et al., [1982]), supplemented with naphthalene or *o*-xylene or different *n*-alkanes in an atmosphere saturated with these compounds, as the only carbon and energy source. Growth of R7 was performed in 100 ml-flasks with 20 ml of M9 mineral medium in presence of *n*-hexane (C6), *n-*octane (C8)*, n-*decane (C10), *n-*dodecane (C12), *n-*hexadecane (C16), *n*-eicosane (C20), *n*-tetracosane (C24)_,_*n*-hexatriacontane (C36) at the concentration of 1 g/l.

**Table 1:** Bacterial strains and plasmids used in this study

**Strain or plasmid**	**Description**	**Reference or source**
**Strains**		
*Rhodococcus opacus* R7	Long-medium-chain *n*-alkane degrader, *alkB*^+^, *nar*^+^, *gen*^+^, *o-xyl*^+^	Di Gennaro et al. [2001]; Di Gennaro et al. [2010]
*Rhodococcus erythropolis* AP	Diesel Fuel degrader	Maffei [2004]
*Escherichia coli* DH5α	d*lac*ZΔM15, *rec*A1, *end*A1, *gyr*A96, *thi*-1, *hsd*R17(rK^−^, mK^+^), *sup*E44, *rel*A1, *deo*R, Δ(*lac*ZYA-*arg*F)U169	Promega
*Escherichia coli* DH5α (pDrive-*alk*R7)	*E. coli* DH5α containing the cloning vector pDrive, *alkB* fragment, Amp^r^	This study
*Escherichia coli* DH5α (pTipQT1-*alk*R7)	*E. coli* DH5α containing the recombinant expression vector pTipQT1, *alkB* fragment, Thio^r^, Amp^r^	This study
*Rhodococcus erythropolis* AP (pTipQT1-*alk*R7)	*R. erythropolis* AP containing the recombinant expression vector pTipQT1, *alkB* fragment, Thio^r^, Tc^r^	This study
**plasmids**		
pDrive	*E. coli* cloning vector, Amp^r^	Qiagen
pDrive-*alk*R7	pDrive containing *alkB* fragment from *R. opacus* R7	This study
pTipQT1	Shuttle-vector Amp^r^*E. coli* – Tc^r^*Rhodococcus* spp.	Nakashima and Tamura [2004b]
pTipQT1-*alk*R7	pTipQT1 containing *alkB* fragment from *R. opacus* R7	This study

*n*-Eicosane (C20), *n*-tetracosane (C24)_,_*n*-hexatriacontane (C36) were added in flasks as hexane solution evaporated over night or as finely ground powder.

*Rhodococcus erythropolis* AP, isolated in our laboratory (CIP 110799) for its ability to grow on diesel fluel (Maffei, [2004]), was maintained on M9 mineral medium in a saturated atmosphere of diesel fuel at 30°C (Table 1). *Escherichia coli* DH5α was grown on Luria-Bertani medium at 37°C. To select *E. coli* and *Rhodococcus* trasformants antibiotics (ampicillin at 100 μg/ml for *E. coli* and tetracycline at 25 μg/ml for *Rhodococcus*) were added in the cultural medium.

Genomic DNA of *R. opacus* R7 was extracted from R7 cells as reported by Di Gennaro et al., [2010]. DNA manipulation, enzymatic digests, ligation and PCR reactions were performed using standard molecular techniques (Sambrook and Russell, [1989]). For recombinant plasmids extraction NucleoSpin Plasmid Kit by Machery and Nagel was used according to the manufacturer’s instruction.

### 2.2 Growth curves and *n*-alkanes biodegradation

Growth experiments on R7 strain were performed in presence of *n*-alkanes (1 g/l) ranging from C6 to C36 in 20 ml of M9 with OD_600_ 0.1.

To determine *R. opacus* R7 *n*-alkanes degradation, the strain was inoculated in 20 ml of M9 with OD_600_ 0.1 and supplemented with 1 g/l of *n*-alkanes (C12, C16, C20, C24). To determine abiotic loss, uninoculated flasks were also prepared. The flasks were incubated at 30°C for a maximum of 72 h. Every 24 h a flask was sacrified to evaluate *n*-alkanes degradation by determination of the residual *n*-alkane by gas chromatography–mass spectrometry (GC-MSD) after extraction with *n*-hexane. Bacterial growth was determined by OD_600_ measurements. When the strain was inoculated on *n*-alkanes with more than 16 carbon atoms, the bacteria formed clumps which included *n*-alkanes (van Beilen et al., [2002]). For optical density measurements, clumps were resuspended by strong agitation.

### 2.3 Chromatographic analyses

Residual *n*-alkanes were extracted from the inoculated and uninoculated flasks using *n*-hexane. It occurred in 20 min by strong manual agitation (in test tubes), after washing of the flasks to take away any residual *n*-alkanes on their wall. The suspension was centrifuged at 4000 rpm for 10 min and after 10 min of settling, 2 ml were drawn from organic phase. It was conserved at −20°C in vial with teflon-coated screw caps. The organic phase extracted was diluted 1:100 for GC injection. Residual *n*-alkanes were analysed by GC-MSD using a Technologies 6890 N Network GC System, interfaced with 5973 Network Mass Selective Detector (MSD) (Agilent Technologies). A ZB-5MS capillary column was used (5% diphenyl-95% dimethylpolysiloxane 60 m x 0.25 mm, 0.25 μm; Alltech).

Analyses were carried out in split-less injection mode using helium as carrier gas at 99.99%. The injector port was set at 250°C. The oven temperature was programmed from 60°C for 3 min, then 15°C min^−1^ to 280°C, holding this temperature for 10 min. Electron impact ionization spectra were obtained at 70 eV, with recording of mass spectra from *m*/*z* 42 to 550 amu, which allows 3.5 scans s^−1^. All analyses were carried out with three replicates, and the mean values obtained are reported.

### 2.4 Analysis of the metabolic intermediates from the *n*-alkane pathway

The metabolic intermediates resulting from incubation of *R. opacus* R7 on C12 were analyzed by GC-MSD after extraction with ethyl acetate. It occurred in 20 min by strong manual agitation (in test tubes) in presence of 1.5 ml HCl 1 M. The suspension was centrifuged at 4000 rpm for 10 min. After centrifugation, 1.5 ml of samples were taken from organic phase and transferred into 1.5 ml vials. For derivatization the solution was stripped under a gentle stream of nitrogen, 10 μL of derivatizing agent TMSI and Pyridine (Supelco) and hexane up to 1 ml were added. The solutions were heated at 70°C for 30 min. The derivatized samples were analyzed by GC-MSD and the extracted samples were conserved at – 20°C in vial with teflon-coated screw caps. The registered mass spectra were compared with those of the spectra library (NIST) of the instrument, and the identification of the metabolites was confirmed by injection and analysis of the corresponding standard compound.

### 2.5 Kinetic modeling

The residual *n*-alkane concentrations as a function of time were modeled according to the Monod-type kinetic models as reported by Simkins and Alexander ([1984]). In particular, considering the substrate scarce water solubility and the biomass initial concentration (growth *viz*. exposition runs), the logistic and first-order kinetic models were applied. For logistic model, the maximum specific growth rate, μ_max_ (h^−1^), was estimated from the microbial growth curve, plotting the natural logarithm of OD_600_ vs time and determining the slope of the linear part of the graph (Zhukov et al., [2007]). The inverse yield parameter, q (ppm OD_600_^−1^) (Simkins and Alexander, [1984]), was estimated as the ratio of the degraded *n*-alkane to OD_600_ increase. A non-linear regression was used for the fitting of the logistic model to the experimental data. The goodness of fit was evaluated on the basis of the determination coefficient, the standard error of the estimated parameter and the p-value.

For first-order kinetic model, the natural logarithm of substrate concentration *vs* time was plotted and the slope of the least-squares-regression line was determined.

### 2.6 Identification and cloning of the *alkB* gene cluster

The isolation of the complete coding region of the *alkB* gene cluster from *R. opacus* R7 was obtained from purified genomic DNA of the strain by PCR-amplification.

To identify the genomic region, two primers (F1, 5′-AAGGCCATGGGGCGTTAGAGCACCGCAGCTAAT-3′ and R1, 5′-ACAGCATATGACCTAGCGGGCGGCCGCGACCCG-3′) were designed on the basis of the more conserved sequences between the *alkB* gene cluster of different bacteria belonging to the *Rhodococcus* genus deposited in Genbank DataBase. It was carried out using the following program: 95°C for 3 min; 95°C for 30 sec, 68°C for 45 sec, 72°C for 5 min, for 35 cycles; and 72°C for 3 min.

DNA fragments were purified from agarose gel by the NucleoSpin Extraction II Kits by Machery and Nagel. The eluted fragment was sequenced by automated sequencing (Eurofins MWG). The nucleotide sequence of the *alkB* gene cluster of *R. opacus* R7 was deposited in the GenBank DataBase (KJ573524).

The 3.0 Kb fragment, containing the *alkB* gene cluster, was then amplified by PCR using the primers *Nco*I-*alk*R7 5′-AAGGCCATGGACGTGACGACGTCGGATATC-3′ and *Nde*I-*alk*R7 5′-ACAGCATATGACCTAGCGGGCGGCCGCGAC-3′ in order to generate the *Nco*I-*Nde*I ends. The 3.0 Kb fragment was cloned as PCR product into the pDrive vector (Qiagen). The ligation mixture was used to transform *E. coli* DH5α by electroporation and the recombinant clones were selected on LB agar supplemented with ampicillin 100 μg/ml and IPTG 1 mM and 5-bromo-4-chloro-3-indolyl-β-D galactopyranoside (X-Gal) 40 μg/ml. White colonies were isolated and plasmid (pDrive-*alk*R7) of the recombinant clones was extracted and verified with digestion *Nco*I/*Nde*I.

### 2.7 Construction of the recombinant strain *R. erythropolis* AP (pTipQT1-*alk*R7) and activity of the *alkB* system

The *alkB* insert was ligated as *Nco*I-*Nde*I fragment into a shuttle-vector *E. coli*-*Rhodococcus*, pTipQT1 (Nakashima and Tamura [2004a]). The ligation mixture was used to transform *E. coli* DH5α by electroporation with standard procedures (Sambrook and Russell, [1989]) and the recombinant clones were selected on LB agar supplemented with ampicillin (100 μg/ml) at 37°C. Ampicillin-resistant clones were selected and the recombinant plasmid (pTipQT1-*alk*R7) was isolated. The same recombinant plasmid was used as shuttle-vector to transform others *Rhodococcus* spp. strains by electroporation. We chose as host *Rhodococcus erythropolis* AP selected in our laboratory (Maffei, [2004]) because the expression of the *Ptip* system efficiency was more performant in *erythropolis* species (Nakashima and Tamura, [2004b]). Plasmids were introduced into strains of *Rhodococcus* spp. by electroporation using a Gene Pulser II (Biorad, Italia) set at 2.50 kV, 600 Ω, 25 μF (Treadway et al., [1999]) in presence of about 1 μg DNA. Immediately after electroporation, 2.5 ml recovery broth (LB medium with 1.8% sucrose) were added and cells were incubated at 30°C for 4 h. Cells were plated on LB supplemented with tetracycline 25 μg/ml and grown at 30°C for 3–4 days. The stability of the recombinant plasmid in the *R. erythropolis* AP was verified after extraction and restriction analysis of the plasmid from all *R. erythropolis* AP (pTipQT1-*alk*R7) clones. All the clones were able to maintain the recombinant plasmid.

Moreover, before biodegradation experiments, a representative amount of colonies of the recombinant strain underwent the same procedure and we verified the maintaining of the plasmid in the following generations.

Recombinant strain *R. erythropolis* AP (pTipQT1-*alk*R7) was used for biodegradation experiments in presence of *n*-alkane C12 to evaluate the activity of the *alkB* system.

### 2.8 Chemicals

All chemicals used were of analytical grade. All organic solvents used were high-performance liquid chromatographic (HPLC) grade supplied from Fluka. Naphtalene, *o*-xylene and *n*–alkanes, 1-dodecanol, 2-dodecanol, dodecanoic acid were supplied from Sigma.

## 3 Results

### 3.1 Growth of *R. opacus* R7 on medium- and long-chain *n*-alkanes

*Rhodococcus opacus* R7 was isolated from a soil contaminated by polycyclic aromatic hydrocarbons, and the strain was able to grow on naphthalene, *o*-xylene (Di Gennaro et al., [2001]; Di Gennaro et al., [2010]) as well as on various medium- and long-chain *n*-alkanes.

The metabolism on *n*-alkanes was investigated in presence of medium- and long-chain *n*-alkanes. Growth of R7 in M9 mineral broth in presence of *n*-hexane (C6), *n*-octane (C8), *n-*decane (C10), *n-*dodecane (C12), *n-*hexadecane (C16), *n-*eicosane (C20), *n-*tetracosane (C24), *n*-hexatriacontane (C36) (1 g/l) supplied as the only carbon and energy source was tested in 96 h. An increasing biomass accumulation was observed by spectrophotometric analysis at OD_600_ every 24 h. *R. opacus* R7 grew with *n*-alkanes ranging in length from C10 to C36, but no growth was observed in the range of C6-C8 *n*-alkanes (Table 2).

**Table 2:** **Degradation of** ***n*-alkanes by** ***Rhodococcus opacus*** **R7**

***n*-Alkane**	**Carbon atoms**	***Rhodococcus opacus*** **R7**
Hexane	6	-
Octane	8	-
Decane	10	+
Dodecane	12	+
Hexadecane	16	+
Eicosane	20	+
Tetracosane	24	+
Hexatriacontane	36	+

-, no growth; +, growth.

Although R7 growth on *n*-alkanes ranging in length from C20 to C36 was difficult to determine, due to the low solubility of solid *n*-alkanes in water, a certain growth was observed around the solid *n*-alkane agglomerates formed and on the wall of the flasks. Before the growth measurement, a strong agitation of the flasks was necessary to observe a homogeneous cells suspension (van Beilen et al., [2002]).

The capacity of *R. opacus* R7 to degrade *n*-alkanes ranging in length from C12 to C24 was investigated as a function of time.

### 3.2 Biodegradation kinetics of medium- and long-chain *n*-alkanes C12-C24

**Figure 1:**
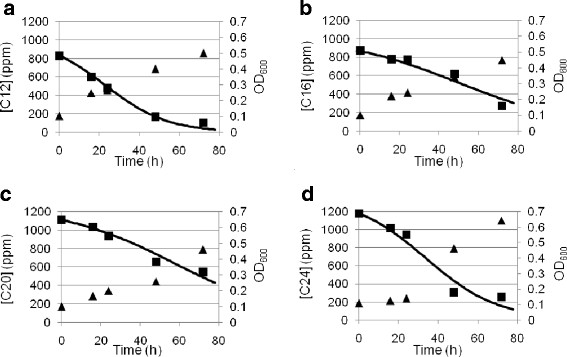
**Kinetic analyses of*n-*dodecane (C12) (a),*n-*hexadecane (C16) (b),*n-*eicosane (C20) (c),*n-*tetracosane (C24) (d) degradation in*R. opacus*R7.** Cells of *R. opacus* R7 were exposed to the *n*-alkane after growth on naphthalene and a flask each day was sacrified for the extraction and the GC-MSD determination of the residual hydrocarbon.

To investigate the capacity of *R. opacus* R7 to degrade *n*-alkanes, kinetic runs were performed in M9 mineral medium supplemented with C12, C16, C20, C24 as the sole carbon and energy source; growth was followed at different times up to 72 h (Figure 1, a, b, c, d). The increase in biomass was quite the same for the four substrates, indeed OD_600_ reached about 0.5-0.7. GC-MSD analyses of *n*-alkane residues were performed after extraction from broths; the results showed that substrate consumption occurred parallel to biomass increase.

The consumption rate of C12 was 88%, of C16 was 69%, of C20 was 51% and of C24 it was 78%. The consumption of C12 was higher than longer *n*-alkanes: the decrement of degradation rate of these hydrocarbons seems to be correlated to the length of the aliphatic chain.

Abiotic controls were performed in presence of each hydrocarbon. The amount of abiotic loss was about 20% for C12, about 24% for C16, about 18% for C20, and about 26% for C24, respectively.

*n*-Alkane residual concentrations as a function of time followed a logistic curve, typical of a biodegradation process where a scarcely soluble organic compound is degraded by a growing microbial culture. From the experimental data treatment, values of the maximum specific growth rate, μ_max_, and of the semisaturation constant, K_S_, were estimated (Table 3). As expected, K_S_ increased from C12 to C24, indicating a decrease in the biodisponibility of the *n*-alkane for the biomass. On the contrary, μ_max_ was almost constant for C12, C16 and C20 and of the order of 0.015 h^−1^, while it approached 0.032 h^−1^ for C24. This last value could indicate that more enzymatic systems were involved in the degradation of C24 or that a different up-take mechanism could be hypothesized.

**Table 3:** **Biodegradation kinetic parameters for** ***n*-alkanes metabolism in** ***R. opacus*** **R7**

**Substrate consumption**			**Cellular growth**		
**Substrate**	**μ**_**max**_**/K**_**S**_**(1/h ppm)**	**R**^**2**^	**μ**_**max**_**(h**^**−1**^**)**	**R**^**2**^	**K**_**S**_**(ppm)**
C12	(6.3 ± 0.3) 10^−5^	0.988	0.013 ± 0.002	0.972	210
C16	(3.1 ± 0.2) 10^−5^	0.938	0.0129 ± 0.0007	0.997	420
C20	(2.7 ± 0.2) 10^−5^	0.952	0.017 ± 0.004	0.956	650
C24	(3.9 ± 0.4) 10^−5^	0.952	0.032 ± 0.009	0.905	810

### 3.3 Metabolic intermediates of *n*-alkane C12 degradation

**Figure 2:**
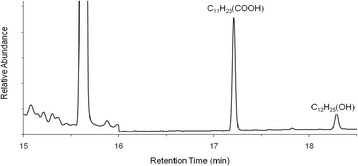
**Cultural broth analysis by GC-MSD.** Cells of *R. opacus* R7 were exposed to *n-*dodecane and at different times, the cultural broth were analysed in GC-MSD. The intermediate metabolites identified are reported in the graph.

Metabolic intermediates of *n*-alkanes in *R. opacus* R7 were determined from cultural broths during the growth on *n-*dodecane as reference substrate, chosen on the basis of the kinetic parameters determined for biodegradation runs. R7 growth on C12 was followed during the exposition to the hydrocarbon as a function of time. At time 0 h, 3 h, 5 h, 8 h and 24 h, culture samples were acidified, extracted, and analyzed by GC-MSD as reported in Material and Methods. Cultural broth analysis showed the presence of 1-dodecanol and the corresponding carboxylic acid (Figure 2) which were identified by comparison with the relative standards. We identified the 1-dodecanol only and not the mixture of 1-dodecanol and 2-dodecanol, as reported in literature for many cases of *Rhodococcus* strains (Whyte et al., [1998]; Whyte et al., [2002]). To confirm these data, both 1-dodecanol and 2-dodecanol were supplied separately as carbon and energy source and growth was observed only in presence of 1-dodecanol and no growth was observed in presence of 2-dodecanol. Moreover, we observed growth when the dodecanoic acid was supplied as the only carbon and energy source.

### 3.4 Identification and sequencing of the *R. opacus* R7 *alkB* gene cluster involved in *n*-alkanes degradation

**Figure 3:**
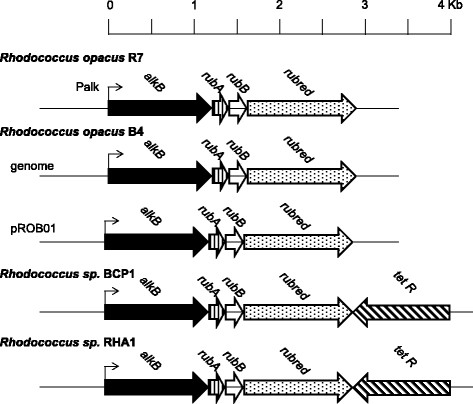
**Gene organization of the** ***alkB*** **gene cluster of** ***R. opacus*** **R7 and comparison with equivalent clusters from other alkane-degrading** ***Rhodococcus*** **bacteria.**

The *alkB* gene cluster from *R. opacus* R7 genomic DNA was identified (Figure 3) and sequenced. This region was isolated by PCR with primers designed on the basis of the alignment of conserved sequences from the *alkB* gene region from different *n*-alkane degrading *Rhodococcus* bacteria. The genomic 3.0 Kb fragment was isolated and sequenced. From the sequence analysis, the covering region contained four consecutive ORFs homologues to the *alkB* gene cluster components: *alkB* encoding for an alkane monooxygenase, *rubA* encoding for a rubredoxin, *rubB* encoding for a second rubredoxin, and *rubred* encoding for a rubredoxin reductase.

These sequences isolated from R7 strain were compared with the ones of other bacteria belonging to the *Rhodococcus* genus. In comparison with *Rhodococcus opacus* B4 (Sameshima et al., [2008]), benzene-tolerant capable of growing on *n*-alkanes, *alkB* gene showed an identity of 94% (the corresponding protein AlkB 91%), *rubA, rubB*, and *rubred* an identity of 90%, 95% and 86% (RubA 91%, RubB 97%, RubRed 85%), respectively. In comparison with *Rhodococcus* sp. BCP1 (Cappelletti et al., [2011]), capable of growing on volatile and medium-chain *n*-alkanes, *alkB* gene presented an identity of 83% (AlkB 80%), *rubA, rubB* and *rubred* an identity of 86%, 84% and 69% (RubA 79%, RubB 84%, RubRed 60%), respectively. Compared to *Rhodococcus jostii* RAH1 (McLeod et al., [2006]), able to degrade polychlorinated biphenyls (PCBs), *alkB* gene presented an identity of 94% (AlkB 91%), *rubA, rubB* and *rubred* an identity of 99%, 94% and 91% (RubA 98%, RubB 97%, RubRed 91%), respectively.

### 3.5 Construction of the recombinant strain *R. erythropolis* AP (pTipQT1-*alk*R7) and activity of the *alkB* system

**Figure 4:**
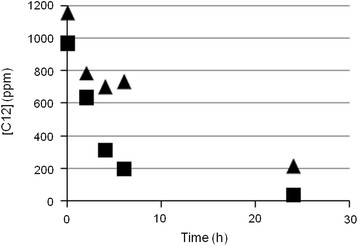
**Kinetic analyses of*n-*dodecane degradation in*R. erytropolis*AP (pTipQT1-*alk*R7) recombinant strain (square in the graph).** Cells of the recombinant strain were grown on rich medium and then, after induction with thiostrepton, were collected and exposed to *n-*dodecane. For comparison, kinetics of *n-*dodecane degradation in *R. erytropolis* AP (triangle in the graph) without the cloned fragment expressing the *alk*B gene, is also showed.

The *alkB* gene cluster was isolated from R7 genomic DNA as PCR product and cloned into the pDrive vector giving the plasmid pDrive-*alk*R7. The region was isolated as *Nco*I/*Nde*I fragment and cloned into the shuttle-vector *E. coli*-*Rhodococcus* pTipQT1. The recombinant plasmid pTipQT1-*alk*R7 was isolated from *E. coli* DH5a and transferred into *Rhodococcus erythropolis* AP, because the *Ptip/regulator* system of the shuttle-vector is more efficient in *Rhodococcus erythropolis* species (Nakashima and Tamura [2004b]). In order to verify the expression of the *alkB* gene cluster under the control of the *Ptip/regulator* system, experiments with resting cells of *Rhodococcus erytropolis* AP(pTipQT1-*alk*R7) exposed to C12 were performed. The expression of the *alk* region was determined comparing the biodegradation kinetics of the recombinant strain *R. erythropolis* AP (pTipQT1-*alk*R7) with the wild type strain *R. erythropolis* AP. Results are reported in Figure 4. The percentage of biodegradation in 6 h was near 80% in the recombinant strain and 37% in the wild type strain. From kinetics analysis we can observe that the initial degradation rate was higher in the recombinant strain with respect to the wild type strain indicating a difference in the activity levels of the *alkB* system. This difference was confirmed by a statistical test for the comparison of the slopes of the regression lines at 95% significance level.

## 4 Discussion

In this paper *Rhodococcus opacus* R7, a strain with a versatile metabolism, was characterized for the ability to degrade variable-chain-length *n*-alkanes.

Many alkane-degrading bacteria have been isolated and the enzyme systems that oxidize *n*-alkanes up to C16 have been characterized (Rojo, [2009]; van Beilen and Funhoff, [2007], Wentzel et al., [2007]). Long-chain *n*-alkanes are more persistent in the environment than the shorter but few data are available in particular concerning the metabolism of these compounds in Gram-positive bacteria (Whyte et al., [2002]; Smits et al., [2002]; Lo Piccolo et al., [2011]).

*R. opacus* R7 showed the ability to grow on medium and long chain *n*-alkanes ranging from C10 to C36. Biodegradation kinetics suggested that R7 strain degraded the *n*-alkanes with a lower number of carbon atoms to a greater extent (88% C12, 69% C16, 51% C20, 78% C24). Moreover, μ_max_ was almost constant for C12, C16 and C20 and about 0.015 h^−1^, while it approached 0.032 h^−1^ for C24. To our knowledge, no literature data are available concerning specific growth rates on single *n*-alkanes, but only Zhukov et al. reported data in presence of a mixture of *n*-alkanes (Zhukov et al., [2007]). The decrement of degradation percentage of these hydrocarbons seems to be inversely correlated to the length of the aliphatic chain as already reported (van Beilen et al., [2003]; Lo Piccolo et al., [2011]). An exception was C24; in fact, when growing on this hydrocarbon, R7 strain showed a higher increase in OD_600_, although the degradation percentage was similar to that observed for C12, and a higher μ_max_. These data suggested that other enzymatic systems could be involved in C24 degradation. Another hypothesis could be a different up-take mechanism, supported by the experimental observation of a massive adhesion of R7 cells to C24 powder (van Beilen et al., [2003]; Rapp et al., [2003]; Lo Piccolo et al., [2011]).

Many strains from different microbial genera are able to grow on *n*-alkanes. Alkanes are usually activated by a terminal oxidation to the corresponding primary alcohol, which is further oxidized by the alcohol and the aldehyde dehydrogenases to the corresponding acid. The resulting fatty acid enters into the beta-oxidation cycle. In some cases *n*-alkanes are metabolized via terminal as well as subterminal oxidation to the corresponding secondary alcohol. The secondary alcohol is converted to the corresponding ketone which is oxidized by a monooxygenase to an ester that is successively hydrolyzed by an esterase to an alcohol and a fatty acid (van Beilen et al., [2003]; Ji et al., [2013]). On the basis of the metabolic intermediates determined by the R7 growth on C12, our data indicated that *R. opacus* R7 metabolizes medium-chain *n*-alkanes by the primary alcohol formation because no trace of the secondary alcohol was found and because the strain is not able to grow on this compound. This result highlights a difference in comparison with many *Rhodococcus* strains in which a mixture of the two alcohols was observed (Whyte et al., [1998] and references therein). In the GC-MSD analysis we also identified the monocarboxylic acid, confirming therefore the activation by a terminal oxidation of the *n*-alkane as reported for *Rhodococcus* sp. MS11 by Rapp et al., [2003].

Moreover, the *alkB* gene cluster from *R. opacus* R7 was isolated and its involvement in the *n*-alkane degradation was studied. In this paper we identified the *alkB* gene cluster including an *alkB* gene, encoding for an alkane hydroxylase, two rubredoxins A and B, and a gene encoding for a rubredoxin reductase that are all required in the catalytic process as electron transfer proteins. Phylogenetic analysis of R7 *alkB* gene cluster showed a significant similarity with the homologous system of *R. opacus* B4 (Sameshima et al., [2008]) and a lower identity with *Rhodococcus* sp. BCP1 (Cappelletti et al., [2011]).

Although four types of *n*-alkane aerobic degradation pathways have been identified to date, the number of alkane hydroxylases that have been isolated, characterized and analyzed by structural biology techniques remains limited. The most widely characterized alkane degradation system is the AlkB of *Pseudomonas putida* GPo1 (Smits et al., [2002]; van Beilen et al., [2003]). Researchers have also isolated and cloned novel genes encoding AlkB from bacteria belonging to *Rhodococcus* genus, such as *Rhodococcus opacus* B4 (Sameshima et al., [2008]) or *Rhodococcus* sp. Q15 (Whyte et al., [2002]), but more information in these bacteria needs to be addressed. Literature data report the involvement of the alkane monoxygenase in the *n*-alkane biodegradation demonstrated by the changing of the GTG coding sequence for the expression in *E. coli* of this enzyme (Sameshima et al., [2008]). Other authors were unable to show function heterologous expression of *alkB* genes in *Pseudomonas* or *E. coli* expression system, principally because the functional expression requires proper synthesis, correct folding, and proper assembly, and these are not always ensured for rhodococcal proteins (Whyte et al., [2002]). On these bases, the *alkB* region identified from *R. opacus* R7 was cloned for the first time into a shuttle-vector *E. coli*-*Rhodococcus* and the alkane hydroxylase activity was evaluated in *R. erythropolis* AP. This strain was chosen not only because it belongs to the *Rhodococcus* genus but also because it belongs to the *erythropolis* species. In fact, the *Ptip/regulator* system of the shuttle-vector showed the best performance when expressed in *erythropolis* species as reported by Nakashima and Tamura, [2004b]. In this way, we were able to overcome difficulties concerning gene expression in heterologous bacteria. Our results showed an increased biodegradation of C12 in the recombinant strain *R. erythropolis* AP (pTipQT1-*alk*R7) in comparison with the wild type strain *R. erythropolis* AP. These data supported the involvement of the *alkB* gene cluster in the *n*-alkane degradation in R7 strain. Considering the biodegradation kinetics for the C12-C24 substrate range, we can hypothesize that for the medium-chain-length *n*-alkanes an *alkB* like system plays the main role, while for *n*-alkanes longer than C20, other alkane hydroxylases could be involved.

## Competing interests

The authors declare that they have no competing interests.

## Authors’ contributions

JZ carried out the molecular biology studies and chemical analysis, participated in the sequence alignment and drafted the manuscript. EC participated in the design of the study and performed kinetic analysis. ML performed statistical data treatments and interpretation of the data. PDG participated in the design of the study and the coordination and helped to draft the manuscript. All authors read and approved the final manuscript.
